# Association between hypertension and the prevalence of liver steatosis and fibrosis

**DOI:** 10.1186/s12902-023-01318-1

**Published:** 2023-04-20

**Authors:** Huanjie Fu, Hao Yu, Yisheng Zhao, Jinhong Chen, Zhichao Liu

**Affiliations:** 1 Department of Cardiovascular, Second Teaching Hospital of Tianjin University of Traditional Chinese Medicine, Tianjin, 300150 China; 2 Intensive Care Unit, Second Teaching Hospital of Tianjin University of Traditional Chinese Medicine, Tianjin, 300150 China; 3 School of Rehabilitation Medicine, Weifang Medical University, Weifang, Shandong 261053 China

**Keywords:** Hypertension, Liver steatosis, Fibrosis, Liver stiffness, Controlled attenuation parameter

## Abstract

**Background:**

Hypertension (HTN) and non-alcoholic fatty liver disease (NAFLD) frequently coexist and share pathophysiological symptoms. Based on the liver stiffness measurement and controlled attenuation parameter obtained by performing liver transient ultrasound elastography (TUE), we determined the relationship between HTN status and the rates of liver steatosis and fibrosis in this study.

**Methods:**

To perform this cross-sectional study, data were obtained from the National Health and Nutrition Examination Survey for 2017-March 2020 Pre-pandemic cycle. The relationship between HTN and the rates of liver steatosis and fibrosis was analyzed by constructing a multivariate logistic regression model. The VCTE was performed using a FibroScan® system (model 502, V2 Touch), and CAP was measured at ≥ 274 dB/m for liver steatosis, and the LSM result (median, ≥ 8 kPa) confirmed fibrosis. We also conducted subgroup analyses based on the age, sex, ethnicity, and body mass index (BMI) of the patients.

**Results:**

In total, 4,705 participants were recruited, including 2,287 participants with HTN and 2,418 without HTN. After adjusting possible confounders, HTN was positively related to the liver steatosis rate (OR = 1.4, 95% CI: 1.1–1.8). Such HTN-associated prevalence was higher among males (OR = 1.6, 95% CI: 1.1–2.2), non-Hispanic African American individuals (OR = 2.1, 95% CI: 1.1–3.7), and participants with BMI ≥ 25 < 30 kg/m^2^ (OR = 1.7, 95% CI: 1.1–2.5). Additionally, HTN was positively associated with the fibrosis rate (OR = 2.0, 95% CI: 1.3–3.0), especially among females (OR = 2.6, 95% CI: 1.3–5.2), among individuals who were 40–59 years old (OR = 2.1, 95% CI: 1.0–4.3), 60–80 years old (OR = 2.4, 95% CI:1.3–4.6), non-Hispanic Caucasian (OR = 2.9, 95% CI: 1.5–5.6), among those with BMI ≥ 25 < 30 kg/m^2^ (OR = 3.0, 95% CI: 1.1–8.2), and those with BMI ≥ 30 kg/m^2^ (OR = 2.1, 95% CI: 1.4–3.2).

**Conclusion:**

The results of this study revealed that HTN status was associated with higher rates of liver steatosis and fibrosis, particularly in subjects with BMI ≥ 25 kg/m^2^. The ethnicity of the participants also had an impact on the relationship.

## Background

Non-alcoholic fatty liver disease (NAFLD) is a common chronic hepatopathy and a major global health concern [1, 2]. It occurs as a result of metabolic syndrome (MetS). NAFLD and hypertension (HTN) have reached epidemic proportions [3]. Some systemic diseases, inflammatory disorders, alcoholism, and infections have a negative impact on the liver and heart. NAFLD is a hepatic manifestation of metabolic disorders that affects the occurrence of cardiovascular diseases (CVDs) [4]. HTN is frequently associated with NAFLD, which affects approximately 40% of the population. NAFLD may increase the likelihood of developing CVDs [5].

Histologically, NAFLD encompasses a disease spectrum ranging from steatosis to mild inflammation (non-alcoholic fatty liver) [6]. NAFLD is divided into two types: non-alcoholic fatty liver (NAFL) and non-alcoholic steatohepatitis (NASH). While NAFL is defined as the presence of ≥ 5% hepatic steatosis without evidence of hepatocyte injury, NASH is defined as hepatic steatosis with accompanying lobular inflammation and hepatocyte injury (e.g., hepatocyte ballooning), with or without fibrosis [7]. NAFLD is linked to metabolic disorders such as dyslipidemia, hypertension, and hyperglycemia. In addition to increased fat content, the accumulation of pancreatic ectopic dysfunctional adipose tissue, which is primarily associated with insulin resistance and beta cell dysfunction, plays an important role in this context [8, 9]. Insulin resistance is accompanied by compensatory persistent hyperinsulinemia, which is critical for establishing and maintaining an unfavorable metabolic milieu (e.g., increased free fatty acid and glucose levels), whereby the prevailing insulin resistance worsens and promotes the development of cardiometabolic disorder [10]. Insulin resistance is associated with dysregulated neurohumoral activation of the renin–angiotensin–aldosterone system, fibrinolytic dysfunction via increased plasminogen activator inhibitor-1 (PAI-1) levels, cardiac autonomic neuropathy, which may promote the development of systolic and diastolic dysfunction or cardiac arrhythmias, endothelial dysfunction, and subsequent hypertension [10–13].

NAFLD is typically diagnosed after liver steatosis is discovered through a liver biopsy, histological analysis, and imaging examinations in the absence of causes of abnormal transaminase values or secondary causes of liver fat accumulation as determined by a medical history or laboratory tests [14, 15]. As a non-invasive imaging method, vibration controlled transient elastography (VCTE) can be used to accurately diagnose liver steatosis and advanced hepatopathy in adults [16]. VCTE was included as a method for detecting liver steatosis and hepatic fibrosis in the most recent cycle of the National Health and Nutrition Examination Survey (NHANES) based on the liver stiffness measurement (LSM) and controlled attenuation parameter (CAP). Using the NHANES database, we examined the relationship between HTN and liver steatosis and fibrosis in adult participants, as measured by CAP and LSM.

## Methods

### Participants

**Fig. 1:**
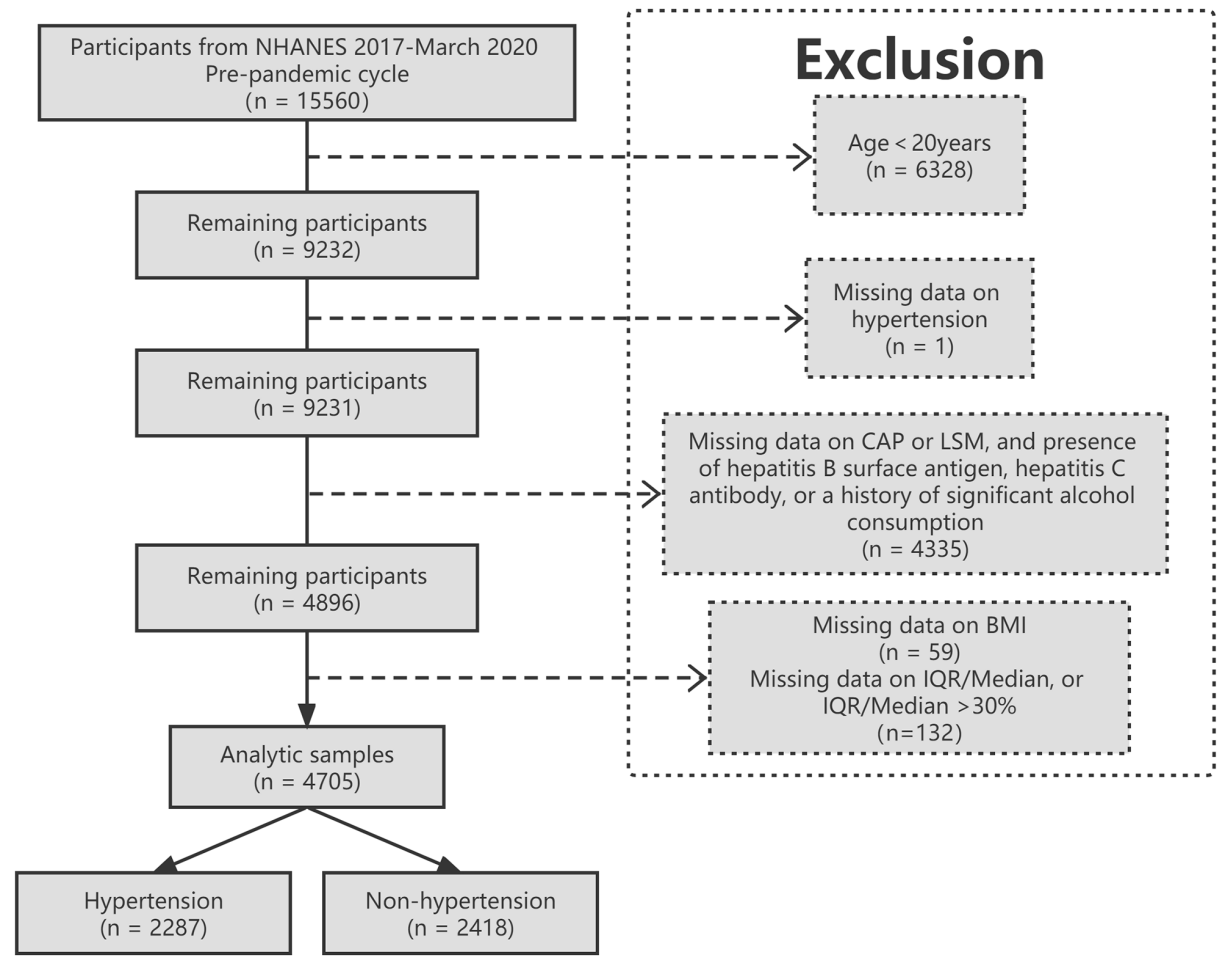
A flow chart describing the sample selection process

This cross-sectional study obtained data from the NHANES database (2017-March 2020 Pre-pandemic cycle). In the NHANES, health data on the US population were collected objectively. The data collection methodology is available on the NHANES website (http://www.cdc.gov/nchs/nhanes.htm) [17]. Of the 9,232 adults (≥ 20 years old) for whom information was available in the database mentioned above, unqualified adults were eliminated as follows, one individual for whom blood pressure values were unavailable; 1,310 for whom LSM or CAP information was unavailable; 3,025 individuals positive for hepatitis C antibody, hepatitis B surface antigen, or with a history of alcoholism (≥ 3 and ≥ 4 drinks/day for women and men, respectively) [18]; 59 individuals for whom information on body mass index (BMI) was unavailable; 132 individuals for whom information on IQR/Median was unavailable, or IQR/Median > 30%. Overall, data on 4,705 participants were included in the analysis. A flow chart describing the outline of our study is presented in Fig. 1.

The National Health and Nutrition Examination Survey serves as the foundation for our survey strategy. All participants provided written informed consent for data collection and information use. Following the guidelines of Strengthening the Reporting of Observational Studies in Epidemiology (STROBE), our study maintained transparency [19, 20].

### Variables in the study

Hypertension status was investigated in this study and was defined based on the following criteria: first, the questionnaire item that stated “ever told you had high blood pressure” represented the self-reported status of HTN; second, mean diastolic pressure > 90 mmHg and mean systolic pressure > 140 mmHg were determined four times; third, the participants with HTN were identified based on their response to the questionnaire item “taking prescribed medication for hypertension”[21]. A FibroScan® system (model 502, V2 Touch) was used for performing VCTE, and CAP was measured at ≥ 274 dB/m for liver steatosis, which indicated steatosis on liver ultrasound [22]. The result of the LSM (median, ≥ 8 kPa) confirmed fibrosis [23], which was measured using the FibroScan® model 502 V2 Touch inVCTE that possessed an extra-large or moderate probe. Besides recording data on clinical and demographic factors, we extracted the data on several variables to be used as covariates, including age, sex, ethnicity, education level, BMI, family income-to-poverty ratio, smoked ≥ 100 cigarettes during the lifetime, and the levels of blood urea nitrogen (BUN), serum glucose, total cholesterol (TC), triglyceride (TG), serum uric acid (SUC), LDL cholesterol, aspartic acid transferase (AST), alanine aminotransferase (ALT), alkaline phosphatase (ALP), gamma-glutamyl transpeptidase (GGT), and glycohemoglobin.

### Statistical analysis

EmpowerStats (X&Y Solutions; Boston, MA) and R (version 3.4.3) were used for conducting statistical analyses, and P < 0.05 represented statistical significance. We constructed a multivariate logistic regression model to determine the association between liver steatosis and fibrosis and HTN status. Three statistical models were considered for data analysis, including model 1 with unadjusted covariates, model 2 with adjusted age, sex, and ethnicity, and model 3 with adjusted covariates shown in Table 1. We also conducted subgroup analyses based on age, sex, ethnicity, and BMI and used Full sample interview weight as the sampling weight for statistical analysis.

**Table 1:** The characteristics of the participants

	Non-hypertension	hypertension	P-value
Age (years)	46.8 ± 16.5	62.1 ± 13.4	< 0.001
Sex (%)			0.584
Men	49.0	49.8	
Women	51.0	50.2	
Race			< 0.001
Non-Hispanic White	33.6	38.0	
Non-Hispanic Black	23.2	31.0	
Mexican American	13.9	8.6	
Other race	29.3	22.4	
Education level			< 0.001
Less than high school	18.5	19.9	
High school	22.7	27.0	
More than high school	58.7	52.8	
Body mass index (kg/m2)	29.0 ± 7.0	31.2 ± 7.3	< 0.001
Ratio of family income to poverty	2.7 ± 1.6	2.6 ± 1.6	0.020
Smoked at least 100 cigarettes in life (%)			< 0.001
Yes	33.5	45.6	
No	66.4	54.4	
Glycohemoglobin (%)	5.7 ± 1.0	6.2 ± 1.2	< 0.001
Serum glucose (mmol/L)	6.0 ± 1.7	6.8 ± 2.5	< 0.001
Alkaline phosphatase (U/L)	76.5 ± 23.8	81.9 ± 26.3	< 0.001
Alanine amino transferase (IU/L)	21.6 ± 15.7	21.7 ± 20.2	0.319
Aspartic acid transferase (IU/L)	21.2 ± 12.1	21.6 ± 15.4	0.379
Gamma-glutamyl transpeptidase (IU/L)	28.1 ± 33.5	32.8 ± 42.5	< 0.001
Serum uric acid (umol/L)	305.8 ± 79.5	340.4 ± 91.5	< 0.001
Blood urea nitrogen (mmol/L)	5.0 ± 1.6	6.0 ± 2.6	< 0.001
Total cholesterol ((mmol/L)	4.9 ± 1.0	4.8 ± 1.1	< 0.001
Triglyceride	1.2 ± 1.4	1.4 ± 1.1	< 0.001
LDL Cholesterol	2.9 ± 0.9	2.8 ± 0.9	< 0.001
Median controlled attenuation parameter (dB/m)	257.1 ± 60.3	279.4 ± 61.3	< 0.001
Liver steatosis (%)			< 0.001
Yes	38.6	53.3	
No	61.4	46.7	
Median liver stiffness (kpa)	5.5 ± 4.4	6.5 ± 5.4	< 0.001
Significant fibrosis (%)			< 0.001
Yes	7.5	15.3	
No	92.5	84.7	

Continuous variables were presented as the mean ± SD, and P-values were determined by performing the Kruskal-Wallis H test (skewed distribution) and one-way ANOVA (normal distribution). Categorical variables were presented as a percentage, and the P-values were determined by performing a Chi-squared test

## Results

As shown in Table 1, the participants were characterized by their HTN status. Of the 4,705 participants enrolled, 2,287 were placed in the HTN group, while the remaining 2,418 participants were placed in the non-HTN group. The HTN patients were older, had higher BMI, higher ALP, GGT, TG, glycohemoglobin, serum glucose, BUN, and uric acid levels, higher LSM and CAP values, and elevated liver steatosis and fibrosis rates than the non-HTN patients, but they had lower TC and LDL cholesterol levels than non-HTN patients.

### Relationship between HTN status and CAP

After adjusting all confounders, our results showed that HTN status positively correlated with CAP (β = 9.1, 95% CI: 4.3–14.0; Table 2). The results of the subgroup analysis showed a positive relationship among women participants (β = 8.8, 95% CI: 1.7–16.0), among men participants (β = 8.5, 95% CI, 1.8–15.2) and also among participants who were 40–59 years old (β = 12.8, 95% CI: 5.4–20.2), non-Hispanic black (β = 17.0, 95% CI: 4.8–29.1), Non-Hispanic White (β = 9.6, 95% CI: 2.0–17.2), and those who had BMI < 25 kg/m^2^ (β = 13.6, 95% CI: 3.0–24.3) and BMI ≥ 25 kg/m^2^ (β = 11.6, 95% CI: 3.7–19.4).

**Table 2:** Relationship between hypertension status and controlled attenuation parameter (dB/m)

	Model 1 β (95% CI, P)	Model 2 β (95% CI, P)	Model 3 β (95% CI, P)
Non-hypertension	Reference	Reference	Reference
hypertension	29.0 (25.5, 32.5) < 0.001	26.9 (23.0, 30.7) < 0.001	9.1 (4.3, 14.0) <0.001
Stratified by sex			
Men (n = 2392)			
Non-hypertension	Reference	Reference	Reference
hypertension	30.2 (25.3, 35.2) < 0.001	30.1 (24.7, 35.4) < 0.001	8.5 (1.8, 15.2) 0.013
Women (n = 2445)			
Non-hypertension	Reference	Reference	Reference
hypertension	27.6 (22.7, 32.4) < 0.001	23.1 (17.6, 28.6) < 0.001	8.8 (1.7, 16.0) 0.015
Stratified by age			
20–39 age group (n = 1049)			
Non-hypertension	Reference	Reference	Reference
hypertension	34.5 (24.4, 44.8) < 0.001	34.9 (24.9, 44.9) < 0.001	4.8 (-7.3, 16.9) 0.434
40–59 age group (n = 1671)			
Non-hypertension	Reference	Reference	Reference
hypertension	31.2 (25.2, 37.2) < 0.001	31.7 (25.7, 37.6) < 0.001	12.8 (5.4, 20.2) < 0.001
60–80 age group (n = 2117)			
Non-hypertension	Reference	Reference	Reference
hypertension	20.3 (14.8, 25.8) < 0.001	21.4 (15.9, 26.9) < 0.001	6.6 (-1.2, 14.4) 0.099
Stratified by race			
Non-Hispanic White (n = 1742)			
Non-hypertension	Reference	Reference	Reference
hypertension	31.6 (25.9, 37.3) < 0.001	28.7 (22.5, 35.0) < 0.001	9.6 (2.0, 17.2) 0.013
Non-Hispanic Black (n = 1312)			
Non-hypertension	Reference	Reference	Reference
hypertension	25.4 (18.7, 32.0) < 0.001	19.3 (11.4, 27.2) < 0.001	17.0 (4.8, 29.1) 0.006
Mexican American (n = 544)			
Non-hypertension	Reference	Reference	Reference
hypertension	16.2 (5.2, 27.1) 0.004	9.1 (-3.0, 21.3) 0.141	-5.5 (-20.7, 9.6) 0.472
Other race (n = 1239)			
Non-hypertension	Reference	Reference	Reference
hypertension	34.0 (27.0, 41.0) < 0.001	30.4 (22.4, 38.4) < 0.001	9.8 (-0.6, 20.3) 0.067
Stratified by body mass index (BMI)			
BMI < 25 (kg/m^2^) (n = 1125)			
Non-hypertension	Reference	Reference	Reference
hypertension	22.0 (16.5, 27.6) < 0.001	14.2 (8.2, 20.3) < 0.001	13.6 (3.0, 24.3) 0.013
BMI ≥ 25, < 30 (kg/m^2^) (n = 1609)			
Non-hypertension	Reference	Reference	Reference
hypertension	10.8 (5.8, 15.9) < 0.001	5.3 (-0.2, 17.3) 0.058	7.6 (-0.9, 16.1) 0.082
BMI ≥ 30 (kg/m^2^) (n = 2103)			
Non-hypertension	Reference	Reference	Reference
hypertension	18.2 (13.3, 23.0) < 0.001	19.8 (14.4, 25.1) < 0.001	11.6 (3.7, 19.4) 0.004

Model 1: No covariate adjustment

Model 2: Adjustment for age, sex, and ethnicity

Model 3: Adjustment for all covariates including age, sex, ethnicity, education, BMI, family income-to-poverty ratio, smoked > 100 cigarettes during the lifetime, BUN, serum glucose, TC, TG, LDL cholesterol, SUC, ALP, ALT, AST, GGT, and glycohemoglobin

### Relationship between HTN status and the prevalence of liver steatosis

As determined by the model adjusted for all covariates (Table 3), HTN status showed a positive relationship with the liver steatosis rate (OR = 1.4, 95% CI: 1.1–1.8). In the subgroup analysis, a positive relationship was found among men (OR = 1.6, 95% CI: 1.1–2.2), and also among participants with BMI ≥ 25 < 30 kg/m^2^ (OR = 1.7, 95% CI: 1.1–2.5), and among those who were non-Hispanic Black (OR = 2.1, 95% CI: 1.1–3.7).

**Table 3:** Relationship between hypertension status and the prevalence of liver steatosis

	Model 1 OR (95% CI, P)	Model 2 OR (95% CI, P)	Model 3 OR (95% CI, P)
Non-hypertension	Reference	Reference	Reference
hypertension	1.8 (1.6, 2.0) < 0.001	1.8 (1.6, 2.1) < 0.001	1.4 (1.1, 1.8) 0.012
Stratified by sex			
Men (n = 2392)			
Non-hypertension	Reference	Reference	Reference
hypertension	1.8 (1.5, 2.1) < 0.001	1.9 (1.5, 2.2) < 0.001	1.6 (1.1, 2.2) 0.015
Women (n = 2445)			
Non-hypertension	Reference	Reference	Reference
hypertension	1.9 (1.6, 2.2) < 0.001	1.7 (1.4, 2.1) < 0.001	1.1 (0.7, 1.6) 0.686
Stratified by age			
20–39 age group (n = 1049)			
Non-hypertension	Reference	Reference	Reference
hypertension	2.1 (1.5, 3.0) < 0.001	2.2 (1.6, 3.2) < 0.001	1.3 (0.6, 2.8) 0.444
40–59 age group (n = 1671)			
Non-hypertension	Reference	Reference	Reference
hypertension	1.9 (1.5, 2.3) < 0.001	2.0 (1.7, 2.5) < 0.001	1.4 (1.0, 2.2) 0.089
60–80 age group (n = 2117)			
Non-hypertension	Reference	Reference	Reference
hypertension	1.3 (1.1, 1.6) 0.002	1.4 (1.2, 1.8) < 0.001	1.3 (0.9, 1.9) 0.180
Stratified by race			
Non-Hispanic White (n = 1742)			
Non-hypertension	Reference	Reference	Reference
hypertension	2.0 (1.6, 2.4) < 0.001	2.0 (1.6, 2.4) < 0.001	1.4 (0.9, 2.1) 0.127
Non-Hispanic Black (n = 1312)			
Non-hypertension	Reference	Reference	Reference
hypertension	2.0 (1.5, 2.5) < 0.001	1.7 (1.3, 2.3) < 0.001	2.1 (1.1, 3.7) 0.016
Mexican American (n = 544)			
Non-hypertension	Reference	Reference	Reference
hypertension	1.5 (1.1, 2.2) 0.025	1.3 (0.9, 2.0) 0.227	0.8 (0.4, 1.9) 0.634
Other race (n = 1239)			
Non-hypertension	Reference	Reference	Reference
hypertension	2.1 (1.7, 2.6) < 0.001	1.9 (1.4, 2.4) < 0.001	1.5 (0.9, 2.5) 0.095
Stratified by body mass index (BMI)			
BMI < 25 (kg/m^2^) (n = 1125)			
Non-hypertension	Reference	Reference	Reference
hypertension	2.4 (1.7, 3.5) < 0.001	1.8 (1.2, 2.7) 0.005	1.9 (0.9, 3.8) 0.084
BMI ≥ 25, < 30 (kg/m^2^) (n = 1609)			
Non-hypertension	Reference	Reference	Reference
hypertension	1.4 (1.1, 1.7) 0.001	1.2 (1.0, 1.5) 0.076	1.7 (1.1, 2.5) 0.012
BMI ≥ 30 (kg/m^2^) (n = 2103)			
Non-hypertension	Reference	Reference	Reference
hypertension	1.4 (1.1, 1.6) 0.001	1.4 (1.1, 1.8) 0.002	1.2 (0.8, 1.8) 0.287

Model 1: No covariate adjustment

Model 2: Adjustment for age, sex, and ethnicity

Model 3: Adjustment for all covariates including age, sex, ethnicity, education, BMI, family income-to-poverty ratio, smoked > 100 cigarettes during the lifetime, BUN, serum glucose, TC, TG, LDL cholesterol, SUC, ALP, ALT, AST, GGT, and glycohemoglobin

### Relationship between the HTN status and LSM

After adjusting the model for all covariates, HTN status was positively associated with LSM (β = 0.5, 95% CI: 0.1–1.0; Table 4). In the subgroup analysis, a positive relationship was found among women (β = 0.4, 95% CI: 0.0–0.8) and also among participants who were 40–59 years old (β = 0.5, 95% CI: 0.0–1.0) and those with BMI ≥ 30 kg/m^2^ (β = 1.2, 95% CI: 0.3–2.0).

**Table 4:** Relationship between hypertension status and the prevalence of liver stiffness (kPa)

	Model 1 β (95% CI, P)	Model 2 β (95% CI, P)	Model 3 β (95% CI, P)
Non-hypertension	Reference	Reference	Reference
hypertension	0.9 (0.6, 1.2) < 0.001	0.9 (0.6, 1.2) < 0.001	0.5 (0.1, 1.0) 0.025
Stratified by sex			
Men (n = 2392)			
Non-hypertension	Reference	Reference	Reference
hypertension	0.7 (0.3, 1.1) 0.002	0.7 (0.3, 1.2) 0.003	0.6 (-0.3, 1.4) 0.180
Women (n = 2445)			
Non-hypertension	Reference	Reference	Reference
hypertension	1.1 (0.8, 1.4) < 0.001	1.0 (0.7, 1.4) < 0.001	0.4 (0.02, 0.8) 0.038
Stratified by age			
20–39 age group (n = 1049)			
Non-hypertension	Reference	Reference	Reference
hypertension	0.9 (0.1, 1.6) 0.026	0.7 (-0.04, 1.5) 0.064	-0.8 (-2.2, 0.6) 0.274
40–59 age group (n = 1671)			
Non-hypertension	Reference	Reference	Reference
hypertension	1.3 (0.9, 1.7) < 0.001	1.3 (0.8, 1.7) < 0.001	0.5 (0.04, 1.0) 0.032
60–80 age group (n = 2117)			
Non-hypertension	Reference	Reference	Reference
hypertension	0.6 (0.1, 1.0) 0.012	0.6 (0.1, 1.0) 0.014	0.4 (-0.3, 1.2) 0.266
Stratified by race			
Non-Hispanic White (n = 1742)			
Non-hypertension	Reference	Reference	Reference
hypertension	0.8 (0.4, 1.3) < 0.001	0.9 (0.4, 1.4) < 0.001	0.6 (-0.2, 1.4) 0.143
Non-Hispanic Black (n = 1312)			
Non-hypertension	Reference	Reference	Reference
hypertension	0.6 (0.2, 1.1) 0.006	0.4 (-0.2, 0.9) 0.181	0.6 (-0.3, 1.5) 0.162
Mexican American (n = 544)			
Non-hypertension	Reference	Reference	Reference
hypertension	0.9 (0.3, 1.4) 0.003	0.2 (-0.4, 0.8) 0.488	-0.3 (-1.1, 0.5) 0.466
Other race (n = 1239)			
Non-hypertension	Reference	Reference	Reference
hypertension	1.5 (1.1, 2.0) < 0.001	1.6 (1.0, 2.1) < 0.001	0.5 (-0.1, 1.0) 0.093
Stratified by body mass index (BMI)			
BMI < 25 (kg/m^2^) (n = 1125)			
Non-hypertension	Reference	Reference	Reference
hypertension	0.4 (0.2, 0.7) 0.001	0.3 (-0.0, 0.6) 0.059	0.1 (-0.5, 0.6) 0.833
BMI ≥ 25, < 30 (kg/m^2^) (n = 1609)			
Non-hypertension	Reference	Reference	Reference
hypertension	0.6 (0.2, 0.9) 0.002	0.2 (-0.2, 0.6) 0.248	0.2 (-0.5, 0.9) 0.553
BMI ≥ 30 (kg/m^2^) (n = 2103)			
Non-hypertension	Reference	Reference	Reference
hypertension	0.7 (0.2, 1.2) 0.007	0.9 (0.3, 1.5) 0.002	1.2 (0.3, 2.0) 0.008

Model 1: No covariate adjustment

Model 2: Adjustment for age, sex, and ethnicity

Model 3: Adjustment for all covariates including age, sex, ethnicity, education, BMI, family income-to-poverty ratio, smoked > 100 cigarettes during the lifetime, BUN, serum glucose, TC, TG, LDL cholesterol, SUC, ALP, ALT, AST, GGT, and glycohemoglobin

### Relationship between HTN status and liver fibrosis

After adjusting the model for all covariates, HTN status showed a positive relationship with liver fibrosis (OR = 2.0, 95% CI: 1.3–3.0) (Table 5). In subgroup analysis, a positive relationship was recorded among women (OR = 2.6, 95% CI: 1.3–5.2) and also among participants who were 40–59 years old (OR = 2.1, 95% CI: 1.0–4.3), 60–80 years old (OR = 2.4, 95% CI: 1.3–4.6), non-Hispanic White (OR = 2.9, 95% CI: 1.5–5.6), and those who had BMI ≥ 30 kg/m^2^ (OR = 2.1, 95% CI: 1.4–3.2) and BMI ≥ 25 < 30 kg/m^2^ (OR = 3.0, 95% CI: 1.1–8.2).

**Table 5:** Relationship between hypertension status and the prevalence of fibrosis

	Model 1 OR (95% CI, P)	Model 2 OR (95% CI, P)	Model 3 OR (95% CI, P)
Non-hypertension	Reference	Reference	Reference
hypertension	2.2 (1.8, 2.7) < 0.001	2.0 (1.6, 2.5) < 0.001	2.0 (1.3, 3.0) 0.001
Stratified by sex			
Men (n = 2392)			
Non-hypertension	Reference	Reference	Reference
hypertension	2.2 (1.7, 2.8) < 0.001	2.0 (1.5, 2.7) < 0.001	1.7 (1.0, 2.9) 0.067
Women (n = 2445)			
Non-hypertension	Reference	Reference	Reference
hypertension	2.3 (1.7, 3.0) < 0.001	2.0 (1.4, 2.8) < 0.001	2.6 (1.3, 5.2) 0.008
Stratified by age			
20–39 age group (n = 1049)			
Non-hypertension	Reference	Reference	Reference
hypertension	2.1 (1.2, 3.7) 0.010	2.0 (1.1, 3.5) 0.023	1.2 (0.4, 3.9) 0.799
40–59 age group (n = 1671)			
Non-hypertension	Reference	Reference	Reference
hypertension	2.2 (1.6, 3.0) < 0.001	2.3 (1.6, 3.1) < 0.001	2.1 (1.0, 4.3) 0.047
60–80 age group (n = 2117)			
Non-hypertension	Reference	Reference	Reference
hypertension	1.8 (1.3, 2.4) < 0.001	1.8 (1.3, 2.5) < 0.001	2.4 (1.3, 4.6) 0.009
Stratified by race			
Non-Hispanic White (n = 1742)			
Non-hypertension	Reference	Reference	Reference
hypertension	2.3 (1.7, 3.2) < 0.001	2.4 (1.7, 3.3) < 0.001	2.9 (1.5, 5.6) 0.002
Non-Hispanic Black (n = 1312)			
Non-hypertension	Reference	Reference	Reference
hypertension	1.9 (1.3, 2.8) < 0.001	1.6 (1.0, 2.4) 0.040	2.3 (0.8, 6.7) 0.125
Mexican American (n = 544)			
Non-hypertension	Reference	Reference	Reference
hypertension	1.6 (0.9, 2.7) 0.089	0.9 (0.5, 1.7) 0.760	0.7 (0.2, 2.8) 0.638
Other race (n = 1239)			
Non-hypertension	Reference	Reference	Reference
hypertension	2.8 (1.9, 4.2) < 0.001	2.8 (1.8, 4.4) < 0.001	2.0 (0.8, 5.1) 0.142
Stratified by body mass index (BMI)			
BMI < 25 (kg/m^2^) (n = 1125)			
Non-hypertension	Reference	Reference	Reference
hypertension	3.1 (1.8, 5.3) < 0.001	2.1 (1.1, 4.1) 0.018	2.9 (0.8, 10.8) 0.110
BMI ≥ 25, < 30 (kg/m^2^) (n = 1609)			
Non-hypertension	Reference	Reference	Reference
hypertension	2.4 (1.6, 3.7) < 0.001	1.7 (1.0, 2.7) 0.032	3.0 (1.1, 8.2) 0.030
BMI ≥ 30 (kg/m^2^) (n = 2103)			
Non-hypertension	Reference	Reference	Reference
hypertension	1.9 (1.6, 2.3) < 0.001	1.6 (1.3, 2.0) < 0.001	2.1 (1.4, 3.2) < 0.001

Model 1: No covariate adjustment

Model 2: Adjustment for age, sex, and ethnicity

Model 3: Adjustment for all covariates including age, sex, ethnicity, education, BMI, family income-to-poverty ratio, smoked > 100 cigarettes during the lifetime, BUN, serum glucose, TC, TG, LDL cholesterol, SUC, ALP, ALT, AST, GGT, and glycohemoglobin

## Discussion

This study looked at the link between HTN status and the prevalence of liver steatosis and fibrosis in adults. Our findings revealed that HTN was linked to an increased risk of liver steatosis, which was more prevalent in men, non-Hispanic Black participants, and those with BMI ≥ 25 < 30 kg/m^2^. HTN status also showed a positive relationship with the prevalence of fibrosis, and it was more prominent among women, non-Hispanic White participants, and participants who were older and those with BMI ≥ 25 kg/m^2^.

Several epidemiological studies have discovered a bidirectional and mutual relationship between HTN and NAFLD, which means that the risk of developing NAFLD increases when people have HTN, and the risk of developing HTN increases when people have NAFLD [24, 25]. Ciardullo et al. conducted a meta-analysis of 11 longitudinal studies. They discovered that NAFLD cases had a 66% higher risk of developing HTN (HR: 1.66, CI: 1.38–2.01), though its prevalence varied with the patients’ age and BMI [26]. Ciardullo et al. found that NAFLD prevalence increased progressively from optimal (16.5%) to normal (34.5%), high normal (39.9%), and elevated blood pressure in another cross-sectional study of 11 489 adults from the 2005 to 2016 National Health and Nutrition Examination Survey (50.2%, P < 0.001). Hypertensive patients also had a higher prevalence of advanced fibrosis (3-9%, based on the specific biomarker used) [27]. Ciardullo et al. also used the National Health and Nutrition Examination Survey data from the 2017–2018 cycle for cross-sectional analysis, and the findings show that blood pressure status was associated with a progressively higher risk of steatosis. In contrast, obesity and diabetes were consistently associated with both steatosis and fibrosis. At the same time, their findings show that there is no significant link between blood pressure and liver fibrosis [28]. This differs slightly from our conclusion, which could be due to differences in inclusion, and exclusion criteria, as well as statistical methods. When compared to NAFLD cases without hypertension, NAFLD cases with HTN have a higher risk of progression [29]. HTN was linked to cardiovascular and all-cause mortality in NAFLD patients in another study (NHANES III) [5].

Non-alcoholic fatty liver disease (NAFLD) is linked to metabolic comorbidities such as obesity [30], type 2 diabetes mellitus (T2DM) [31], or dyslipidemia [32], and thus may be a hepatic manifestation of a metabolic disorder. NAFLD can causeclinical or subclinical CVDs in addition to hepatic morbidity and mortality. Patients with NAFLD have an increased risk of HTN, cardiac arrhythmias, cardiomyopathy, and coronary heart disease (CHD), as well as increased cardiovascular morbidity and mortality in the clinic. Patients with advanced NAFLD, such as those with non-alcoholic steatohepatitis (NASH) and advanced fibrosis, are at the highest risk of developing CVDs [7].

A liver biopsy is the most accurate method of diagnosing and staging the severity of NASH. However, it is costly and invasive and may result in complications and interobserver variability among various pathological characteristics. Several non-invasive methods for diagnosing NASH and staging liver fibrosis have been proposed, including TE, which can be used to estimate liver stiffness as a surrogate for liver fibrosis [33, 34]. An NHANES study found that HTN is independently related to NAFLD fibrosis; however, race-dependent differences exist [35]. Our findings also revealed that HTN status was significantly related to CAP or LSM among individuals of a specific ethnicity but not to CAP or LSM in the Mexican-American population.

Non-alcoholic fatty liver disease (NALFD) might develop into cirrhosis, which might include complications such as malignant tumors and is associated with CVDs or metabolic diseases [36, 37]. Genetic factors with susceptibility to NAFLD have an important effect on inflammation and lipid metabolism, thus affecting hypertension status [38–40]. Metabolic dysfunction is strongly related to the complicated mechanism involving the development of NAFLD; therefore, NAFLD might be called metabolic dysfunction-associated fatty liver disease (MAFLD). In this condition, metabolic dysfunction includes obesity, T2DM, hypertension, metabolic syndrome, and dyslipidemia [40–42]. NAFLD is an underdiagnosed metabolic disorder that is linked to a high prevalence of prehypertension and hypertension [43]. HTN and NAFLD share risk factors and have synergistic effects on the development and complications of the disorders. Therefore, routine screening for HTN in NAFLD cases and people undergoing lifestyle changes, such as physical activity and dietary changes, is required to prevent and manage HTN and NAFLD [44].

Our research had some limitations. First, because this was a cross-sectional study, causal relationships could not be established. Second, the participants’ blood pressures were measured at a single point in time, which may not accurately reflect blood pressure variation. Thus, hypertension was defined using a variety of criteria. Third, the CAP value used to define liver steatosis in various studies based on the NAHENS 2017–2018 database was inconsistent with the LSM value used to define obvious [23, 45, 46]. Therefore, the sensitivity and specificity of the VCTE test varied depending on the cut-off value. Fourth, different measurements were obtained due to the different FibroScan probes [47, 48]. Elastography, on the other hand, was performed by qualified and trained technicians following specific protocols [49]. Finally, self-reported confounders may have caused individual bias, which can be reduced by using NHANES data extracted by trained personnel using appropriate procedures.

## Conclusion

Overall, HTN was associated with higher rates of liver steatosis and fibrosis, which was stronger in subjects with BMI ≥ 25 kg/m^2^ and was influenced by the participants’ ethnicity. Our findings suggested that screening for HTN in NAFLD patients could aid in preventing and managing both HTN and NAFLD.

Continuous variables were presented as the mean ± SD, and P-values were determined by performing the Kruskal-Wallis H test (skewed distribution) and one-way ANOVA (normal distribution). Categorical variables were presented as a percentage, and the P-values were determined by performing a Chi-squared test.

## Data Availability

All data utilized or analyzed in this work can be obtained from NHANES website (http://www.cdc.gov/nchs/nhanes.htm).

## List of abbreviations

NAFLD: Non-alcoholic fatty liver disease
HTN: Hypertension
TE: Transient elastography
NHANES: National Health and Nutrition Examination Survey
CAP: Controlled attenuation parameter
LSM: Liver stiffness measurement
HbA1c: Glycohemoglobin
BMI: Body mass index
BUN: Blood urea nitrogen
GGT: Gamma-glutamyl transpeptidase
ALT: Alanine amino transferase
ALP: Alkaline phosphatase.
